# Contrast sensitivity function under three light conditions in patients with type 1 diabetes mellitus without retinopathy: a cross-sectional, case–control study

**DOI:** 10.1007/s00417-023-06057-6

**Published:** 2023-04-11

**Authors:** María-Carmen Silva-Viguera, Marta C. García-Romera, María-José Bautista-Llamas

**Affiliations:** Department of Physics of Condensed Matter, Optics Area Vision Research Group (CIVIUS), University of Seville, Seville, Spain

**Keywords:** Contrast sensitivity, Diabetic complications, Diabetic retinopathy, Type 1 diabetes mellitus, Visual disorders

## Abstract

**Purpose:**

To determine whether patients with type 1 diabetes mellitus (T1DM), without any sign of diabetic retinopathy, have any alteration in Contrast Sensitivity Function (CSF), in relation to patients without this disease, and whether CSF assessment in three different light conditions can be an effective test in the early detection of diabetic retinopathy.

**Methods:**

A prospective, cross-sectional, case-control study was preformed including 80 patients (40 with T1DM without diabetic retinopathy and 40 controls) between 11 and 47 years old. CSF was assessed at four spatial frequencies (3, 6, 12 and 18 cycles/degree) using the CSV-1000E test, under three light conditions: high (550 lx), medium (200 lx) and low (< 2 lx).

**Results:**

A lower CSF in the T1DM group was found at the three light conditions studied. The most spatial frequency affected was 18 cpd, 0.08 log units (*p =* 0.048) in high, 0.10 log units (*p =* 0.010) in medium (*p =* 0.010) and 0.16 log units (*p* < 0.001) in low-light conditions in mean CS values. The least spatial frequency affected was 3 cpd (*p* > 0.05 in all three light conditions).

**Conclusion:**

Patients with T1DM, without diabetic retinopathy, presented a loss of CS to sine-wave gratings, with respect to people with the same characteristics without the disease, mainly at medium and high frequencies, and in medium and low-light conditions.


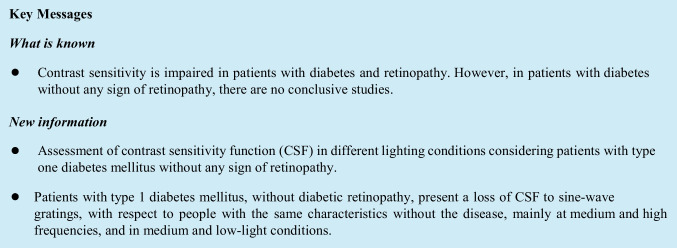


## Introduction

Diabetes mellitus (DM) is a chronic, metabolic disease in which there is high blood-glucose levels, which could cause serious injury of the heart, blood vessels, eyes, kidneys, and nerves over time. According to the World Health Organization and International Diabetes Federation (IDF), DM is one of the fastest-growing global health emergencies in the 21st century, being considered a public health problem [1, 2]. The onset of type 1 DM (T1DM) is associated with younger people, and it is characterized by the lack of production of insulin due to an immunologic system attack [3, 4].

In the evolution of DM there could be several structures affected, such as modification in blood vessels [5] or neuropathies [6] that generally lead to diabetic foot, renal disease or eye complications, [7] mainly diabetic retinopathy. The above being said, it is known that in the retina, even with early diagnosis, at the time of DM detection, it could be observed that microangiopathy affects small retinal vessels, with a major vascular permeability, ocular haemorrhage, and lipid exudates [5]. Thus, the importance of analysing signs in preclinical diabetes aids the possibility of sooner diagnosis.

The study of visual disturbances using non-invasive tests in the DM preclinical phase leads us to consider examining visual function through analysing visual acuity (VA) or contrast sensitivity function (CSF) measurements. VA might be defined as the ability to discern between separate points and identify shapes, CS is the minor contrast detected or the possibility of differentiating two points which are close, and CSF is the contrast detection threshold measured in four or more circles with bands of varying spatial frequencies. Whilst in the standard Snellen test the only aspect considered is the letter size with a high contrast (black-on-white), observing the ability to resolve fine detail, CS measurement allows us to analyse the capacity to detect low-contrast objects of various sizes. Therefore, the qualitative information offered by CSF values concerning visual function is higher than that of VA. It is common for a person to reach the smallest letter on the card on a VA test but report visual impairment or discomfort that often corresponds to reduced CS [8]. Besides, CSF is affected by ambient lighting changes. Generally, high ambient lighting leads to better CSF. However, pupil contraction in higher light conditions must be considered. This reduces aberrations as well as diminishes foveal illumination [9]. Hence, to make a complete diagnosis of a patient's visual function, different lighting and viewing conditions must be considered [10]. CSF values can be achieved using many procedures and tests, such as measurements by variable contrast optotype (Pelli-Robson, Rabin Test or Mars Letter Test) [11] or sine-wave gratings of different spatial frequencies (CSV-1000 test or OPTIC 6500 test) [12, 13]. CSF values could be different with age and the use of several drugs [14].

The impairment of CS in people with DM has been studied by several authors. A review on psychophysical assessments of diabetic retinopathy (DR) included some studies on CS and people with DM with and without diabetic retinopathy, concluding that CS is a more sensitive test than VA for identifying early changes in visual function and signs of neurodegeneration in people with diabetes [15]. However, most of these studies were performed in patients with type 2 DM (T2DM) or do not distinguish the type of DM. Since the course of diabetes is different in patients with T1DM and T2DM, it is advisable to study them separately [16]. A systematic review on the assessment of CS in the early detection of DR has recently been published. This systematic review included 21 comparative cross-sectional studies, published in the last 11 years. It was observed that patients with DM and DR showed a deterioration of CS, however, those without signs of retinopathy showed a lower CSF than control patients, not always significantly, and without unanimity in the affected frequencies. Furthermore, there are few studies on CSF in T1DM without DR and CSF measurements were not performed in different light conditions [17].

The purpose of this study was to determine whether patients with T1DM without any sign of DR have any alteration in CSF, in relation to patients without this disease, and whether CSF assessment in three different light conditions can be an effective test in the early detection of DR.

## Material and methods

### Study design and ethics

A prospective, cross-sectional, case-control study was conducted between May 2021, and October 2021, at the Optometry facilities, the Faculty of Pharmacy, Seville University. The study was approved by the Ethics Committee of the Regional Government (Junta de Andalucía) (code 0997-M1-18) and its development ensured compliance with the ethical principles for medical research contained in the Declaration of Helsinki, as well as the treatment and confidentiality of patients. Adults signed an informed consent form after the nature and consequences of the study were explained to them, and minors under 18 years of age, gave verbal consent, and the informed consent was signed by parents or legal guardians.

### Patients

This study included patients with T1DM and patients without T1DM, as a control group. For the selection of patients with T1DM, a proposal to participate in the study was sent by e-mail to the Seville Diabetic Association (ANADIS). The controls were selected from the educational community of the Seville University Faculty of Pharmacy. Inclusion and exclusion criteria were the same for all participants (T1DM and controls).

The following participants were included: (1) Patients aged between 11 and 50, (2) patients with distance and near corrected monocular visual acuity (VA) better than 0.10 LogMAR and refractive error (RE) less than 4,00 D (sphere) and 3,00 D (cylinder), (3) patients diagnosed with T1DM by an endocrinology physician at least 3 years earlier, insulin-dependent and without any sign of DR on fundus imaging, according to the Early Treatment of Diabetic Retinopathy Study (ETDRS) [18].

The following participants were excluded: (1) Patients with systemic or ocular disease (corneal opacities, cataracts, glaucoma, diabetic retinopathy and other retinal disease) or visual impairment (amblyopia, strabismus, anisometropia) that could affect the results of the visual examination, (2) patients with pharmacological prescriptions that could affect the results of the visual examination, (3) patients with previous eye surgery (including those with intraocular lenses), (4) patients with intraocular pressure higher than 21 mmHg, (5) pregnant or nursing women, (6) patients without T1DM who had capillary glycosylated haemoglobin (HbA1c) higher than 5.6% [19].

### Measurements and study protocol

A data collection sheet was used for each participant which included personal data, medical history as well as optometric data.

All participants underwent anterior pole evaluation using a slit lamp (Topcon SL-6E, Japan) and posterior pole evaluation by non-mydriatic retinography (CSO Non-mydriatic Fundus Camera Cobra HD, Italy). All fundus images were reviewed by two of the authors independently to check the fundus image for signs of DR, i.e. no microaneurysms or any other features of DR, as described in the ETDR Study [18]. In addition, an optometric evaluation in which refractive error was determined to establish current spectacle correction, VA for long and short distance, prior to CS recording were performed, and intraocular pressure using a non-contact tonometer (Topcon CT-800, Japan) and HbA1c level, using the Cobas b-101 analyser (Roche Diagnostic) were determined. On verifying that participant met the established inclusion and exclusion criteria, CS was evaluated.

Objective refraction was performed by static retinoscopy (Welch Allyn retinoscope, Mexico) followed by subjective refraction (Essilor MPH100E S/N 000104 phoropter) to obtain the refractive error of each eye (sphere and cylinder), noting the VA with the new correction. For statistical analysis of the data, the spherical equivalent (SE) was used.

VA in monocular and binocular long distance was tested by an ETDRS optotype in a backlit light box (Lighthouse Low Vision Products, Long Island City, NY, USA) (85.0 cd/m^2^) located at 4 m. For short distance, the monocular and binocular VA was measured with an ETDRS chart at 40 cm. To prevent patients from memorizing the letters and committing bias, patients were asked to read with their right eye and binocularly from left to right and with their left eye from right to left [20]. The VA was collected in LogMAR format.

CSF was assessed using CSV-1000 test (Vector Vision, Inc., Ohio, USA) at 2.50 m under three different light conditions: (1) High: room lights fully on (550 lx incident at the patient's eye position), (2) Medium: room lights slightly on (200 lx incident at the patient's eye position) and (3) Low: room lights off (< 2 lx incident at the patient's eye position due only to test´s illumination). Room lighting was measured with a Lux LCD Illuminance Meter (Precision Vision®, Woodstock, IL, USA). Between each measurement, a period of 15 min was allowed for the patient to adapt to the new light condition. All participants wore test glasses with their long-distance correction in place before proceeding with the CS measurement. Each eye was tested separately. The CSV-1000 test consists of four sine-wave gratings, with light and dark zones corresponding to four different spatial frequencies: 3, 6, 12 and 18 cycles/degree (cpd). Each grid is presented with eight contrast levels in steps of 0.17 log units for contrast levels 1–3 and 0.15 log units for 4–8 [21]. The test has its own lighting system with a luminance of 85 cd/m^2^. Patients were instructed to choose between two targets exposed in two rows (one with the light and dark banded grid and the other with solid grey). The results were collected on the data record sheet provided by the manufacturer, noting the minimum level of contrast that the patient was able to perceive for the four spatial frequencies. Values were transposed to log units by the table listed on the company's website (http://www.vectorvision.com/csv1000-norms/) for curve fitting and analysis [22].

The same material was used for every patient, and the same order was followed in all tests. All patients were evaluated by appointment and by the same optometrist. All patients were advised not to wear contact lenses within 48 h prior to measurements.

### Statistical analysis

Statistical analysis of the data was performed using IBM SPSS® Statistics 26 for Windows program (IBM Corporation, Armonk, NY). The normality of the variables was analysed using the Shapiro-Wilk test. Descriptive analysis was performed using the frequency (percentage), the mean ± standard deviation (SD) and median (interquartile range). The differences between the two groups were analysed using the independent *t* test for variables with normal distribution, the Mann-Whitney U test for variables without normal distribution and the Chi-square (*χ*^2^) test for qualitative variables. Friedman test was used to assess the differences among the three light conditions, and a Bonferroni correction was applied to address the problem of multiple comparisons. In the comparison between monocular variables, because no significant differences were observed in the measurements between the right and left eye, only data from one eye per patient were considered for statistical analysis, choosing either the right or left eye by means of simple computer-generated random numbers. The relationship between the variables considered was assessed using the Spearman Rho test. For all comparisons, a *p* value less than 0.05 was considered statistically significant.

The sample size was determined in the GRAMMO® calculator (Institut Municipal d’Investigació Mèdica, Barcelona, Spain. Version 8.0) based on the result of CS measurement at 6 cpd frequency under low-light conditions. The common SD is assumed to be 0.2 for the group with T1DM based on the results obtained by Safi et al. [21]. Accepting an alpha risk of 0.05 and a beta risk of 0.2 in a two-sided test, a minimum of 38 subjects are necessary in each group to recognise a statistically significant difference greater than or equal to 0.13 log units in the CS values.

## Results

### Demographics and clinical characteristics

The sample population consisted of 80 patients, 40 patients with T1DM (T1DM group) and 40 patients without T1DM (control group). The descriptive characteristics of the participants by study group, as well as the *p* value resulting from the comparison between the T1DM and control groups, are presented in Table 1. The T1DM and control groups were similar in terms of age, sex, refraction, distance or near visual acuity, and intraocular pressure. The only statistically significant difference found between the two groups was the levels of HbA1c (*t* = 18.594, *p* < 0.001).

**Table 1:** Demographics and clinical characteristics of participants in the study and *p* value resulting from the comparison between the two groups

Variables (Units)	T1DM Group (*n* = 40)	Control Group (*n* = 40)	*p* value
Age (years)	27.28 ± 12.3 (11 to 47)	24.40 ± 10.6 (11 to 46)	0.376
Male, n (%) Female, n (%)	12 (30) 28 (70)	17 (42) 23 (58)	0.245
HbA1c (%)	7.44 ± 0.81 (5.4 to 9.4)	5.00 ± 0.19 (4.6 to 5.4)	< 0.001^a^
Diabetes duration (years)	12.3 ± 8.7 (3 to 35)	–-	–-
IOP (mmHg)	15.6 ± 2.4 (10 to 20)	15.9 ± 2.3 (12 to 20)	0.8001
Refractive Error (SE) (Dioptres)	-0.28 ± 1.49 (-3.50 to 3.50)	-0.41 ± 1.41 (-3.50 to 3.75)	0.4301
Monocular Distance VA (LogMAR)	-0.08 ± 0.06 (-0.25 to 0.04)	-0.06 ± 0.07 (-0.20 to 0.10)	0.1201
Binocular Distance VA (LogMAR)	-0.12 ± 0.06 (-0.24 to 0.00)	-0.11 ± 0.07 (-0.24 to 0.02)	0.2081
Monocular Near VA (LogMAR)	-0.05 ± 0.05 (-0.10 to 0.06)	-0.07 ± 0.05 (-0.16 to 0.04)	0.1261
Binocular Near VA (LogMAR)	-0.08 ± 0.04 (-0.18 to 0.08)	-0.09 ± 0.05 (-0.18 to 0.00)	0.0641

D: dioptres, HbA1c: capillary glycosylated haemoglobin, IOP: intraocular pressure, SE: spherical equivalent, T1DM: type 1 diabetes mellitus, VA: visual acuity, %: percentage

Data are presented as mean ± standard deviation (range)

^a^ Statistically differences thought independent* t* test

### Contrast sensitivity assessment results

The results corresponding to the evaluation of CSF in the three different room-light conditions per group and the statistical significance (*p* value) obtained in the comparison between both groups, are shown in Table 2.

**Table 2:** Contrast sensitivity results at the three light conditions studied in the type 1 diabetes mellitus and control groups, and *p* value resulting from the comparison between the two groups

SF (cpd)	T1DM Group (*n* = 40)		Control Group (*n* = 40)		*p* value
	Mean ± SD	Median (IQR)	Mean ± SD	Median (IQR)	
*High-light condition (550 lx)*					
3	1.86 ± 0.17	1.78 (1.78, 2.08)	1.92 ± 0.12	1.93 (1.78, 2.08)	0.059
6	2.08 ± 0.18	2.14 (1.99, 2.29)	2.15 ± 0.14	2.14 (2.14, 2.29)	0.135
12	1.80 ± 0.16	1.84 (1.69, 1.99)	1.86 ± 0.14	1.84 (1.77, 1.99)	0.120
18	1.32 ± 0.18	1.25 (1.25, 1.55)	1.40 ± 0.15	1.40 (1.25, 1.55)	0.048^a^
*Medium-light condition (200 lx)*					
3	1.87 ± 0.17	1.78 (1.78, 2.08)	1.92 ± 0.11	1.93 (1.78, 2.08)	0.133
6	2.04 ± 0.19	1.99 (1.84, 2.14)	2.14 ± 0.14	2.14 (1.99, 2.29)	0.017^a^
12	1.75 ± 0.19	1.69 (1.54, 1.99)	1.87 ± 0.14	1.84 (1.69, 1.99)	0.004^a^
18	1.30 ± 0.19	1.25 (1.10, 1.40)	1.40 ± 0.15	1.40 (1.25, 1.55)	0.010^a^
*Low-light condition (*< *2 lx)*					
3	1.79 ± 0.17	1.78 (1.78, 1.89)	1.85 ± 0.14	1.78 (1.78, 1.93)	0.114
6	2.02 ± 0.19	1.99 (1.84, 2.14)	2.15 ± 0.15	2.14 (1.99, 2.29)	0.002^a^
12	1.71 ± 0.23	1.77 (1.54, 1.99)	1.81 ± 0.17	1.84 (1.69, 1.99)	0.063
18	1.27 ± 0.17	1.25 (1.25, 1.40)	1.43 ± 0.15	1.55 (1.25, 1.55)	< 0.001^a^

Cpd: cycles per degree, IQR: interquartile range, SD: standard deviation, SF: spatial frequency, T1DM: type 1 diabetes mellitus

^a^ Statistically significant differences by Mann-Whitney U test

**Fig. 1:**
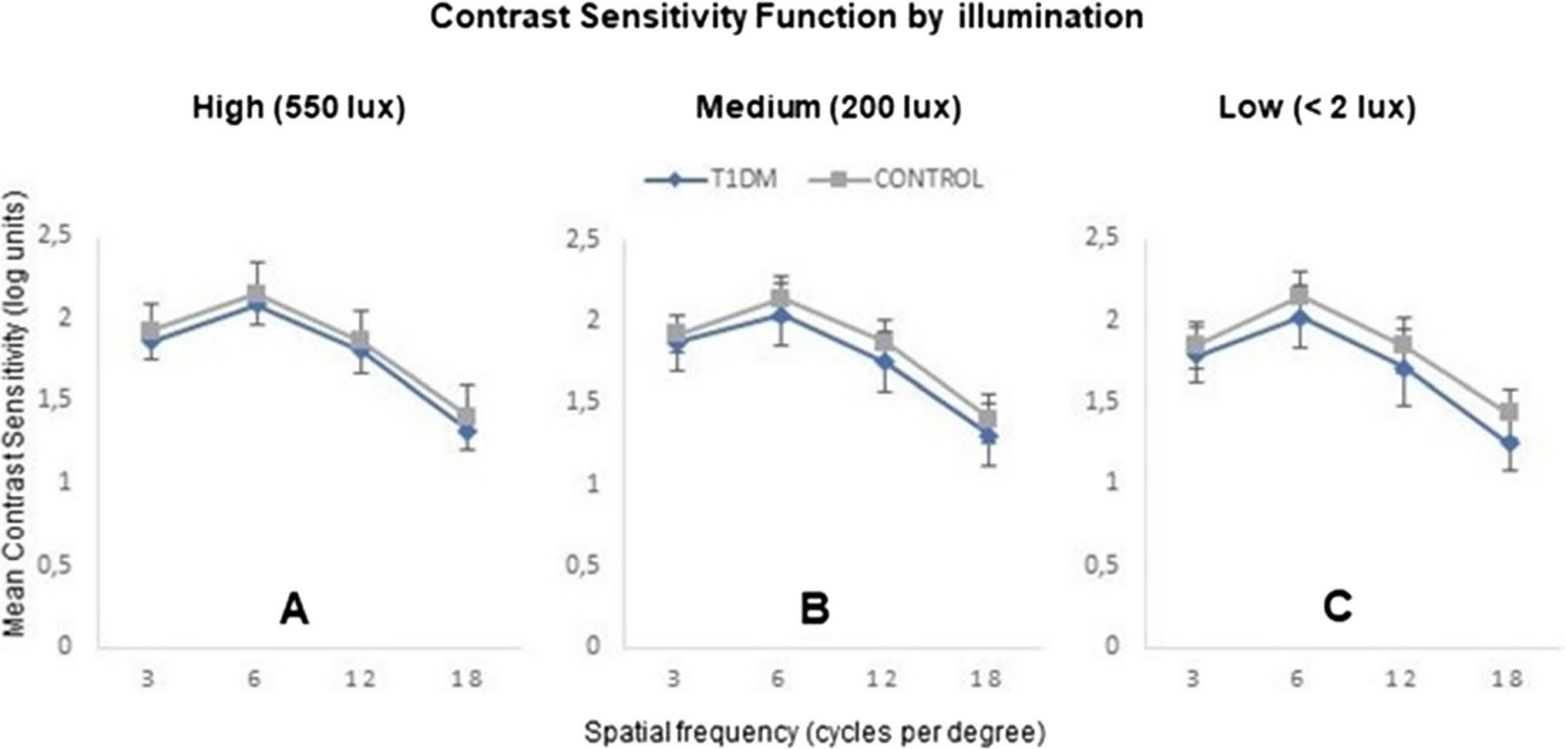
Contrast Sensitivity Function in three light conditions in type 1 diabetes mellitus (T1DM) and control groups. (**A)** High: room lights fully on (550 lx incidents at the patient's eye position). (**B**) Medium: room lights slightly on (200 lx incidents at the patient's eye position). (**C**) Low: room lights off (< 2 lx incident at the patient's eye position due to test illumination only). Error bars are ± Standard deviation

Lower CS values were obtained in the T1DM group with respect to the control group in high-light conditions, with a difference in the mean CS value between groups of 0.07 log units (U = 566.000, *p =* 0.023), ranging from 0.06 log units at 3 cpd (*p* > 0.05) and 12 cpd (*p* > 0.05) to 0.08 log units at 18 cpd (U = 602.500, *p =* 0.048) (Fig. 1A).

In the medium-light condition, the T1DM group presented a mean decrease in CS with respect to the control group of 0.10 log units (U = 492.500, *p =* 0.003), ranging from 0.05 log units at 3 cpd (*p* > 0.05) to 0.12 log units at 12 cpd (U = 509.500, *p =* 0.004) (Fig. 1B).

Mean CS values were also lower in the T1DM group at low-light condition. The mean difference in CS between groups was 0.11 log units (U = 474.500, *p =* 0.002), ranging from 0.06 log units at 6 cpd (U = 491.500, *p =* 0.002) to 0.16 log units at 18 cpd (U = 412.500, *p* < 0.001) (Fig. 1C).

In the comparison of the CS results between the three light conditions (high, medium, and low) by study group (T1DM and control), a statistically significant difference was found among the three light conditions in the T1DM group at 18 cpd (*p =* 0.042). However, in the pairwise comparisons, the adjusted *p* value using the Bonferroni correction for several tests was not significant for any of the comparisons (*p* > 0.05). The difference in the mean value of all frequencies between the three illuminations was statistically significant (*p =* 0.003) in the T1DM group, corresponding to the high-light and low-light condition comparison (*p =* 0.011 after Bonferroni adjustment). Regarding the control group, a statistically significant difference was obtained between the three illuminations at 3 and 12 cpd (*p =* 0.001 and *p =* 0.038 respectively), although none of the pairwise comparisons were statistically significant after adjusting the *p* value by Bonferroni correction (*p* > 0.05).

In the comparison of the difference in CS values between the T1DM group and the control group as a function of room lighting (high, medium, and low), no statistically significant differences were obtained among the three light conditions in any of the SF studied (*p* > 0.05).

### Correlation analysis of age, HbA1c and diabetes duration with contrast sensitivity

The results of the correlations between CS and age, at the three lighting conditions are shown in Table 3. A mild-moderate inverse statistically significant correlation between CS and age was obtained for almost all SF under all three lighting conditions. Regarding the correlations between CS and HbA1c, a weak inverse statistically significant correlation was obtained at low-light conditions at 6 cpd (*ρ* = -0,243, *p =* 0,030) and at medium-light conditions at 12 cpd (*ρ* = -0.241, *p =* 0,032), but no statistically significant correlations were found at high-lighting conditions.

**Table 3:** Statistically significant correlations between contrast sensitivity and age, and between contrast sensitivity and duration of diabetes in the three lighting conditions studied

SF (cpd)	Correlations CS- Age		Correlations CS- DM duration	
	Spearman's coefficient	*p* value	Spearman's coefficient	*p* value
*High-light condition (550 lx)*				
3	-0.379	0.001	-0.261	0.019
6	-0.383	< 0.001	-0.228	0.042
12	-0.382	< 0.001	–	–
18	–	–	-0.252	0.024
*Medium-light condition (200 lx)*				
3	-0.305	0.006	-0.331	0.003
6	-0.357	0.001	-0.384	< 0.001
12	-0.395	< 0.001	-0.338	0.002
18	–	–	-0.331	0.003
*Low-light condition (*< *2 lx)*				
3	-0.512	< 0.001	-0.229	0.041
6	-0.418	< 0.001	-0.360	0.001
12	-0.399	< 0.001	-0.299	0.007
18	-0.260	0.020	-0.471	< 0.001

CS: contrast sensitivity; cpd: cycles per degree; DM: diabetes mellitus; SF: spatial frequency

In terms of the correlations between CSF and diabetes duration, considering diabetes duration 0 in control-group patients, a slight inverse correlation was obtained with low-light conditions at all spatial frequencies (Table 3). However, when correlations between CS and HbA1c and between CS and diabetes duration were studied only in the T1DM group, no significant correlations were obtained between HbA1c and CS. Regarding diabetes duration and CS in the T1DM group, a statistically significant correlation was found at 12 cpd at low-light conditions (*ρ* = -0.345, *p =* 0.029).

## Discussion

This study analysed the CSF in three different light conditions, in people with T1DM who had no signs of DR, and in people with similar characteristics (age, sex, RE and VA) without this disease. The results showed a generalized decrease in CS in the T1DM group with respect to the control group in the three light conditions investigated, despite these patients having good VA (*p* > 0.05). The most affected SF was found to be the highest (18 cpd), with a statistically significant difference between groups of 0.08 log units at high, 0.10 log units at medium and 0.16 log units at low-light conditions in the mean CS values, and the lowest decrease in CS at the lowest SF (3 cpd) (*p* > 0.05).

Other authors have evaluated CS in people with T1DM [17, 23] with different tests [24, 25]. Georgakopoulos [26] using the CSV-1000 test, showed an impairment of CS in all spatial frequencies in patients with T1DM compared to controls, with losses between 0.10 and 0.13 log units, which were statistically significant, as in our study, although, the light conditions were not specified. Also, Krasny [23] included patients with non-proliferative diabetic retinopathy. The CSV-1000 was also used in the studies by Heravian [27] and Safi [21] however, these studies mainly involved people with T2DM, with non-proliferative diabetic retinopathy and with a higher mean age (51.0 ± 9.95 and 48 ± 6 years, respectively), without specifying the light conditions. Only Safi evaluated CS in two light conditions (500 lx and < 2 lx). Our results agree with this study in the lower CS in the diabetes group compared to the control group, but not in the SF in question, as Safi showed statistically significant differences in all SF at < 2 lx, and at 500 lx in all SF except at 3 cpd [21].

As shown in Fig. 1, the differences in CS between T1DM and non-T1DM participants are more pronounced in low-light conditions (0.11 log units in mean across SF) than in high-light conditions (0.07 log units in mean across SF), consistent with the results of Safi et al. [21]. This finding could be related, in addition to the aberrations generated by a larger pupil size in low illumination, to the illuminated area of the retina (influence of rod and cone activation) [17]. On the other hand, we did not find statistically significant differences among the three light conditions (high, medium, and low) in CS loss in people with T1DM. This is probably because the CSV-1000 test is not sensitive for detecting minimal changes [28], and the possible test-retest variability [29–31], therefore, it would be advisable to use a more sensitive test in a future study.

In other studies, on CS in different illuminations, instead of ambient illumination, as in this study, the luminance of the test stimulus was modified by changes in the luminance of the screen and/or the use of neutral density filters to modify foveal information [32], however, the aim of this study was to observe in which room illumination it could be useful to assess CS for early detection of DR. It would be interesting to see whether changes in test luminance would cause greater effects than those observed.

Some authors indicate that in patients with T1DM there may be neuronal changes in the retina, in addition to and independently of vascular changes [25, 33, 34], Studies in patients with diabetes found a thinning of the nerve fibre layer thickness, more consistent with loss of retinal ganglion cells and their axons [35]. Moreover, several investigations have described a thinning of the central retinal thickness in patients with pre-diabetes and diabetes without any sign of DR [36–38]. CS represents the quality of central function and is largely mediated by ganglion cells. This would imply an impairment of CSF in people with DM and the potential of its evaluation in screening for early detection of complications [15]. Future research will be necessary to establish reference threshold values for the measurement of CS in patients with T1DM [39].

Electroretinography (ERG) and optical coherence tomography (OCT) have also been investigated as clinical tests for the early detection of DR, with satisfactory results in both [36, 40–46]. This corroborates the existence of vascular and/or neuronal changes prior to the signs visible in fundus images, which can be detected using these techniques. However, in our opinion, CSF assessment could be a good option for monitoring the patient's visual quality and follow-up. It is a less costly test than those proposed and simple to perform.

Regarding the relationship between CS and other variables, we found a statistically significant correlation with age, as has been reported in other studies [14]. No statistically significant correlation between CS and HbA1c in the T1DM group was found, coinciding with the results of Safi et al. [21]. The correlation obtained between CS and HbA1c in the total sample is not comparable, since Safi did not measure HbA1c in the control group. Geogakopoulos et al. [26], obtained a significant correlation of HbA1c and CS at 3 cpd, but without specifying the ambient light condition under which it was measured.

As for the correlation between CS and diabetes duration, an inverse correlation was obtained at 12 cpd in low-light conditions, while the other authors found no relationship. This may be due to the fact that in Georgakopoulos' study, patients were younger and had a shorter duration of diabetes (5.9 ± 3.8 years) [26], and in Heravian's study, although they had a shorter duration (5.9 ± 3.3 years), they were mostly T2DM and older [27]. Therefore, the relationship between greater CSF impairment with longer duration of T1DM diabetes is not clear, as the patient's glycaemic control also plays a role [17], nor is it clear with higher HbA1c levels. Several investigations have confirmed the influence of glucose variability on the development of diabetic microangiopathy, with a different behaviour between people with T1DM and T2DM. In the case of people with T1DM, an increased risk of retinopathy has been related, not only to high HbA1c values, but also to a high variability in these values [16].

We consider the sample size to be a limitation in this study. Indeed, a larger number of patients would have allowed us to study the behaviour of CS as a function of age and to observe whether it is the same in minors and adults. On the other hand, due to the cross-sectional design of the study, it is not possible to establish a relationship between T1D and CSF, and long-term results would be needed, thus, these results should be considered with caution. Future longitudinal studies in which CSF is followed up, would be necessary to corroborate these results and to establish the relationship with blood glucose.

This lower CS in people with T1DM, although it may have been statistically significant in some frequencies, depending on the illumination, was not clinically significant, as it was not enough to be able to discriminate when the vascular signs of DR would start, therefore, the follow-up of these patients over time is fundamental for early diagnosis.

## Conclusions

The results of this study show that people with T1DM, without signs of diabetic retinopathy, presented a loss of CS to sine-wave gratings, with respect to people with the same characteristics without this disease, mainly in medium and high frequency, and in medium and low-light conditions. This loss of CS is related to age, however, its relationship with HbA1c values and the duration of diabetes is not so clear. Future longitudinal studies will be necessary to establish a threshold for detection of diabetic retinopathy by CSF assessment.

## Data Availability

The data showed in this research are available on request from the corresponding author. The data are not publicly available due to their containing information that could compromise the privacy of research participants.
